# Counselling in Fetal Medicine: Complications of Monochorionic Diamniotic Twin Pregnancies

**DOI:** 10.3390/jcm13237295

**Published:** 2024-11-30

**Authors:** Sara Sorrenti, Asma Khalil, Francesco D’Antonio, Valentina D’Ambrosio, Fabrizio Zullo, Elena D’Alberti, Martina Derme, Ilenia Mappa, Daniele Di Mascio, Giuseppe Rizzo, Antonella Giancotti

**Affiliations:** 1 Department of Maternal and Child Health and Urological Sciences, Sapienza University of Rome, 00185 Roma, Italy; sara.sorrenti@uniroma1.it (S.S.); fabrizio.zullo@uniroma1.it (F.Z.); elena.dalberti@uniroma1.it (E.D.); martina.derme@uniroma1.it (M.D.); daniele.dimascio@uniroma1.it (D.D.M.); giuseppe.rizzo@uniroma1.it (G.R.); antonella.giancotti@uniroma1.it (A.G.); 2 Vascular Biology Research Centre, Molecular and Clinical Sciences Research Institute, St George’s University of London, London SW17 0RE, UK; asmakhalil79@googlemail.com; 3 Fetal Medicine Unit, St George’s Hospital, London SW17 0QT, UK; 4 Center for Fetal Care and High-Risk Pregnancy, Department of Obstetrics and Gynecology, University of Chieti, 66013 Chieti, Italy; dantoniofra@gmail.com; 5 Department of Obstetrics and Gynecology, University of Rome Tor Vergata, 00133 Roma, Italy; mappa.ile@gmail.com

**Keywords:** twin pregnancy, monochorionic, diamniotic, complicated pregnancy, MCDA

## Abstract

Twin pregnancies are at increased risk of morbidity and mortality compared to singletons. Among all twins, monochorionic pregnancies are at higher risk of specific and non-specific complications compared to dichorionic pregnancies. Therefore, it is of great importance to properly counsel future parents with monochorionic pregnancies regarding the risks of adverse outcomes and the modalities of monitoring and intervention of the potential complications. Conditions related to the monochorionicity include twin-to-twin transfusion syndrome (TTTS), twin reversed arterial perfusion sequence (TRAP), and twin anemia polycythemia syndrome (TAPS); other complications include selective fetal growth restriction (sFGR) and congenital anomalies. This review aims to summarize the information available in the current literature regarding the complications in monochorionic diamniotic twin pregnancies, including outcomes and guideline recommendations about the clinical surveillance, management, and timing of interventions of these conditions that should be included in counselling in routine clinical practice.

## 1. Introduction

Monochorionic twin pregnancies (MC) account for 15% of all twin pregnancies. In the majority of cases, they result from the fertilization of one oocyte that splits during the early stages of replication of the blastocyst, causing the sharing of the placenta with two different amniotic sacs (13–14%) of twin pregnancies, when splitting occurs in the first 3 to 8 days, or with the sharing of the same amniotic sac (<1% of cases, when splitting occurs approximately after 8 days) [1].

Monochorionic pregnancies are considered high-risk pregnancies compared to dichorionic twins and singleton pregnancies. The peculiar vascular architecture characterized by the presence of vascular anastomosis can be responsible for the imbalance between the circulations of the two fetuses that can lead to the complications that uniquely occur in these pregnancies [2]. These include twin–twin transfusion syndrome (TTTS), selective intrauterine growth restriction (sIUGR), twin anemia polycythemia sequence (TAPS), and twin reversed arterial perfusion (TRAP) sequence, and they require specific management [3,4,5,6]. Moreover, monochorionic pregnancies are at increased risk for major structural anomalies, particularly congenital heart diseases [7,8].

The screening for complications in monochorionic twin pregnancies plays an essential role; the early diagnosis of any of these conditions leads to early treatment, and it is associated with better outcomes in terms of perinatal morbidity and mortality [5,9].

It is mandatory to offer proper counselling to patients to inform them about the risks and the possible outcomes of these high-risk pregnancies from the first trimester.

## 2. Screening of Complications

The ultrasound surveillance in monochorionic pregnancies is different from that offered for dichorionic or singleton pregnancies because of the possible complications that may occur and rapidly evolve to severe stages, requiring prompt intervention.

The International Society of Ultrasound in Obstetrics and Gynaecology (ISUOG) guidelines recommend performing an ultrasound assessment every two weeks from 16 weeks onward in all MC twin pregnancies to evaluate the parameters that may be indicative of complications (Table 1) [5,9]. Several studies have been conducted in order to identify early sonographic features helping to differentiate between those pregnancies at high risk for complications from those at low risk, since this intensive ultrasound surveillance has a big impact on patients and costs. A cohort study of 177 monochorionic diamniotic pregnancies conducted by Mackie et al. reported that the risk of adverse obstetric outcomes was significantly higher in the case of increased intertwin nuchal translucency (NT) discordance (adjusted OR 1.03 [95% CI 1.01, 1.06]) or in the case of intertwin crown-rump length (CRL) discordance (adjusted OR 1.17 [95% CI 1.07, 1.29]) [10]. In contrast, other studies showed the intertwin CRL discordance to be poorly predictive for adverse fetal outcomes such as fetal loss, perinatal loss, and birthweight discordance [11].

A recent meta-analysis investigated the ability of first-trimester pregnancy-related factors (ultrasound measurements, maternal characteristics, biomarkers) to predict complications in monochorionic twin pregnancies; although NT ≥ 95th percentile in one twin, CRL discordance ≥ 10% and maternal ethnicity were found to be moderately associated with the increased risk of TTTS, and the authors concluded that none of these demonstrated a good diagnostic accuracy for TTTS, sFGR, or intrauterine fetal death [12]. Lewi et al. proposed a double-step approach to identify high-risk pregnancies (in the first trimester and at the 16-week scans), but they correctly identified only 58% of complications, therefore concluding that MC pregnancies remain to a large extent unpredictable [13].

At present, the best approach remains to offer intensive monitoring with an ultrasound every two weeks from 16 weeks onward looking at several parameters in order to identify common complications, as expressed in Table 1 [5]. It is important to counsel patients with monochorionic pregnancies regarding these complications that occur in approximately one third of all cases.

## 3. Congenital Anomalies

As already mentioned, monochorionic twins have an increased risk of developing congenital anomalies, which is nearly twice as high as the risk observed in dichorionic pregnancies and six times higher than that observed in singleton pregnancies [14]. The most common anomalies are congenital heart defects (CHDs) but also anomalies that involve the central nervous system, the genitourinary tract, the musculoskeletal system, and the digestive syndrome, where chromosomal anomalies may occur [14]. Despite the assumption that in the great majority of cases, monochorionic pregnancies share the same genotype, congenital anomalies may be discordant in the two fetuses.

A recent meta-analysis about congenital heart defects in monochorionic twins reported that the prevalence of CHD overall was 59.3 per 1000 live births (95% CI: 50.5–69.4) [15]. The most frequent congenital heart defects reported by the meta-analysis were ventricular septal defects (25.9 per 1000 live births), right ventricular outflow tract obstruction (22.3 per 1000 live births), atrial septal defects (13.6 per 1000 live births), coarctation of the aorta (2.1 per 1000 live births), aortic stenosis (2.6 per 1000 live births), tetralogy of Fallot (0.9 per 1000 live births), and transposition of the great arteries (0.9 per 1000 live births) [15]. These last two conditions had a similar prevalence in singleton pregnancies (0.3 per 1000 live births) [15].

The risk of developing heart anomalies is higher in monochorionic twins who develop TTTS, especially involving the right outflow tract; in particular, the meta-analysis stated that the risk of being diagnosed with a cardiac defect in twins with TTTS was more than two times higher than in twins without TTTS (RR 2.4; 95% CI: 1.6–3.5) [15]. The prevalence of congenital heart defects in TTTS twins was also described by Springer et al. [8], demonstrating that the overall prevalence of CHD in this cohort was 8.9%, with a different distribution according to the Quintero stage groups (30% of cases in stage I, 40% in stage II, 21% in stage III, 1% in stage VI, 7% in stage V). The authors reported a higher prevalence of biventricular hypertrophy and right ventricular hypertrophy in pregnancies complicated by TTTS compared to uncomplicated monochorionic twin pregnancies (10% vs. 0.3% *p* < 0.001 and 3.6% vs. 0.5% *p* 0.007) [8]. Also, cardiomegaly was found to be more frequent in twins with TTTS (3.6% vs. 1% *p* 0.01) [8].

The prenatal detection rate of cardiac anomalies was 48% in the latter study [8]; other authors reported a sensibility of 88.8% of fetal echocardiography in a tertiary referral centre in the prenatal detection of cardiac defects in twin pregnancies [16].

On the other hand, in twin pregnancies complicated by TTTS, the detection rate of congenital cardiac defects is reported to be lower (42.9% in the recipient twin and 16.7% in the donor twin) [17]. This may be explained by the impairment in the imaging acquisition because of polyhydramnios, the excessive movements of the recipient, and the oligoanhydramnios of the donor.

ISUOG guidelines regarding fetal echocardiography recommend offering fetal echocardiography to monochorionic pregnancies if resources allow for it in different clinical settings [18].

Congenital anomalies in monochorionic twins may also involve the cerebral nervous system, including ventriculomegaly, lissencephaly, non-lissencephalic cortical dysplasia, and heterotopia, which may be diagnosed in one or both twins [19]. Moreover, a discordant diagnosis of acrania or anencephaly may develop in monochorionic pregnancies as well as dichorionic twin pregnancies [20].

Other congenital anomalies that can be diagnosed in monochorionic pregnancies involve the genitourinary system (unilateral or bilateral renal agenesis, renal dysplasias, renal cortical necrosis, or horseshoe kidney) or other body systems [19].

At present, the standard for anatomy assessment and the detection of structural anomalies is the 20-week scan, performed according to ISUOG standard guidelines [5,21]. However, recent evidence supports the value of the first-trimester scan in twin pregnancies to assess early fetal anatomy. A meta-analysis conducted by D’Antonio et al. about the early detection of fetal congenital anomalies in monochorionic and dichorionic twins reported a detection rate of 27.3% (95% confidence interval, 15.0–42.8) in the first trimester scan, particularly for cranial, midline brain, and abdominal wall defects [22]. Similarly, a recent large cohort study about the role of the first-trimester scan in diagnosing structural anomalies in the first trimester was conducted in a tertiary centre, and the authors concluded that the rate of anomalies was higher in monochorionic pregnancies compared to dichorionic pregnancies (2.8% vs. 1.3%), as well as the proportion of anomalies detected in the first-trimester scan (52.6% in MC vs. 27.1% in DC) [23].

In the case of discordant congenital anomalies, selective termination of the pregnancy should be discussed with the future parents as an option. In monochorionic twins, it is performed by umbilical cord occlusion, preferably after 18 weeks of pregnancy, in contrast with what is described for dichorionic pregnancies, as the survival rates of the co-twin seem to be higher at that gestational age compared to earlier procedures [24,25]. The cord occlusion may be performed by different techniques, such as bipolar cord coagulation, radiofrequency ablation (RFA), the ligation of the cord vessels, or laser cord coagulation [24]. There are no conclusive data about the optimal method of selective termination in monochorionic twins; however, a review on this topic stated that the highest survival rates of the co-twin were reached by radiofrequency ablation and bipolar cord coagulation [24]. The treatment of choice should be individualized for every single case and widely discussed with the parents. For instance, in cases of oligohydramnios, the use of RFA might be preferred because of the technical difficulties related to the use of fetoscopy. In addition, RFA might be safely performed at the earliest gestational ages.

## 4. Twin-to-Twin Transfusion Syndrome (TTTS)

### 4.1. Pathophysiology

This condition affects 10–15% of monochorionic diamniotic pregnancies, and it is the cause of increased morbidity and mortality in these pregnancies. The etiology of TTTS is the imbalanced blood flow through abnormal vascular anastomoses in the shared placenta. Arterio-arterial and veno-venous anastomoses are physiological in monochorionic twins, and they usually have a bidirectional blood flow; arterio-venous anastomosis, instead, can have a unidirectional blood flow, which can determine the exchange of blood flow from one twin (donor) to the other (recipient) through their placental districts [3,26]. This exchange is the cause of the volume overload in the recipient twin, who becomes hypervolemic, and the volume depletion in the donor, who becomes hypovolemic.

### 4.2. Diagnosis

The diagnosis of TTTS is performed by ultrasound after 16 weeks when there are oligohydramnios (deep vertical pocket (DVP) < 2 cm) of one twin (donor) associated with polyhydramnios of the other twin (recipient) (deep vertical pocket >8 cm before 20 weeks or >10 cm after 20 weeks) [5,27]. However, some cases of TTTS may present without the aforementioned diagnostic criteria, especially before 18 weeks, when the fetal urinary output is still not the primary source of amniotic fluid and the deep vertical pocket of the recipient twin may not reach 8 cm [28]. Before 18 weeks of pregnancy, the cut-off for the DVP should be lowered to 6 cm in order to include all cases that present with the clinical characteristics of a twin-to-twin transfusion syndrome but do not reach the standard diagnostic criteria [28].

### 4.3. Staging

The staging of TTTS that is used in clinical practice is the Quintero staging (Table 2) [29]. A recent meta-analysis reported that in stage I and stage II, the survival of at least one twin was 86.9% (95% CI, 84.0–89.7%) and 85.0% (95% CI, 79.1–90.1%), respectively; in stage III, the survival of at least one twin was 81.5% (95% CI, 76.6–86.0%); in stage IV, it was 82.8% (95% CI, 73.6–90.4%); and in stage V, the survival of the remaining twin was reported as 54.6% (95% CI, 24.8–82.6%) [30]. The rate of no surviving twins was 11.8% in stage I, 15% in stage II, 18.6% in stage III, 17.2% in stage IV, and 45.4% in stage V [30]. The same study reported that respiratory and neurological morbidities were lower in stage I and they increased in stages II–IV, but this finding might also be related to the role of iatrogenic prematurity in these neonates [30].

Another classification system developed for TTTS is the CHOP (Children’s Hospital of Philadelphia) score, based on echocardiographic features such as ventricular structural and functional characteristics and valvular function in the recipient, as well as umbilical artery flow in the donor [31]. In TTTS, in fact, some cardiac functional abnormalities may occur in the recipient because of the volume overload, including pulmonary stenosis, which is often the cause of hypertrophy of the right ventricle, biventricular hypertrophy (61%), diastolic dysfunction and subsequent systolic dysfunction in the right (50%) and in the left ventricle (58%), and atrioventricular valve regurgitation (21%) [32]. A validation study was conducted to evaluate this classification method and a significant relationship between the CHOP score and the Quintero staging system was found, but it did not show prognostic value in terms of pregnancy outcomes [33]. At present, the CHOP score is not used in routine clinical practice.

### 4.4. Management

The management strategies in TTTS include expectant management, amnioreduction in the recipients’ sac, fetoscopic laser ablation of placental anastomosis, selective reduction in pregnancy, and intentional septostomy to equalize amniotic fluids. Amnioreduction may be performed once or several times, from 14 weeks of pregnancy onward; it may reduce the amniotic pressure due to excessive amniotic fluid excretion in the recipient twin, improving the placental blood flow and reducing the risk of preterm birth due to polyhydramnios [32]. The risks associated with this procedure include the preterm rupture of membranes, placental abruption, preterm labour, and fetal death [32,34].

Intentional septostomy is not recommended because no significant advantages were observed because of the high risk of membrane disruption and for the consequences of the functional state of monoamniotic twin pregnancy [32].

Selective reduction in pregnancy may be considered, and it is performed via the cord occlusion of the donor twin using radiofrequency ablation or cord coagulation [32].

The gold standard for fetal treatment in TTTS is the fetoscopic laser ablation of placental anastomosis, performed with equatorial dichorionization (Solomon technique). The purpose of this intervention is to interrupt the pathological arterio-venous anastomosis, including all non-visible anastomosis, functionally separating the two parts of the placenta. Complications associated with the laser procedure comprise chorioamnionitis, placental abruption, premature rupture of membranes, preterm labour and delivery, or the fetal demise of one or both twins [32].

A multicenter randomized trial published by Senat et al. [35] compared the outcomes of amnioreduction versus fetoscopic laser therapy in fetuses between 15 and 26 weeks with severe TTTS; the laser technique was associated with higher survival of at least one twin, a later gestational age at delivery of the surviving twin, and better long-term neurological outcomes [35,36]. Similar results were reported by a Cochrane review on the topic, with a significant reduction in the risk of overall death (RR 0.81, 95% CI 0.65–1), perinatal death (RR 0.59, 95% CI 0.40–0.87), and neonatal death (RR 0.29, 95% CI 0.14–0.61) with laser photocoagulation compared to amnioreduction [37].

The treatment option for stages II-IV of TTTS diagnosed before 26 weeks is laser photocoagulation. Some evidence also suggests that this treatment may be beneficial after 26 weeks if resources are available [38,39].

Moreover, a recent meta-analysis has investigated the role of laser coagulation in TTTS diagnosed in early pregnancy (before 18 weeks), and it found no differences in the overall survival in cases treated before or after 18 weeks; however, an earlier gestational age at delivery was observed in cases treated before 18 weeks [40].

On the other hand, the management of stage I TTTS has been widely debated. A meta-analysis about the different management options in Quintero stage I fetuses reported that the survival of at least one fetus was similar in the expectant management group, in the amnioreduction group, and in the laser group (87% (95% CI 69–98%), 86% (95% CI 76–94%), 81% (95% CI 69–90%), respectively) [41].

Similarly, a recent multicentric randomized trial compared stage I TTTS cases between 16 and 26 weeks of pregnancy treated with laser ablation within 72 h from diagnosis or expectant management [42]. The results showed similar survival rates without neurological compromise at 6 months, with 77% in the expectant group and 78% in the immediate surgery group (*p* 0.88) [42]. However, the progression rate from the expectant management to the need of rescue laser treatment was 59%, mainly due to disease progression, maternal symptoms of polyhydramnios, or shortening cervical length [42].

In conclusion, conservative management with close surveillance may be a reasonable option in stage I cases; in case of progression to a higher Quintero stage or in case of worsened conditions, such as worsened polyhydramnios, maternal discomfort due to polyhydramnios, or shortening cervical length, laser therapy should be immediately considered [5].

Providing proper counselling to the parents is mandatory, especially in stage I, when the expectant management can be considered as a safe option. Up to date, there are no predictors of disease progression at stage I that will determine the need for rescue surgery, so the counselling should be based on the available evidence in the current literature and personalized for each case and to parental wishes.

### 4.5. Follow-Up

ISUOG guidelines suggest weekly monitoring of Doppler velocimetry and amniotic fluid in pregnancies complicated by TTTS, in both stage I managed expectantly or after laser therapy for the first two weeks after the procedure [5]; no randomized trials have been published on this topic. The recurrence rate of the disease after laser ablation is 14%; it may be caused by some persistent or revascularized anastomosis, which are less frequent with the Solomon technique [43]. Also, deep placental anastomoses may not be treated with laser dichorionization [44].

### 4.6. Long-Term Neurodevelopmental Sequelae

Twin pregnancies complicated by TTTS are at increased risk of developing brain abnormalities; neurodevelopmental delay has been reported in up to 23% of cases treated by laser ablation at long-term follow-up (2 years) [45,46]. This risk appears to be similar in the donor and in the recipient survivors, and different lesions may occur, such as intraventricular hemorrhage, cystic periventricular leukomalacia, migration anomalies, ventriculomegaly, hydrocephaly, and infarction [47,48]. A recent meta-analysis reported the overall rate of brain damage in TTTS fetuses treated with laser ablation as 2.2% (1.03% in the recipient and 0.82% in the donor twin), with the most common lesions being of ischemic type (30.4%, 95% CI, 19.1–43.0) [49]. Risk factors for the development of brain injuries include a late gestational age at laser treatment, recurrent TTTS, and the occurrence of TAPS [47].

Prenatal MRI in the third trimester may be useful in addition to ultrasound neurosonography for detecting and characterizing brain abnormalities [47].

### 4.7. Time of Delivery

Up to date, no evidence exists about the timing of delivery in monochorionic twin pregnancies complicated by TTTS. The existing studies about the cohort of patients treated with laser report the gestational age at delivery to be around 33–34 weeks [35,50]. Since prematurity is an additional risk factor for adverse outcomes in these neonates, the administration of a single course of antenatal steroids for lung maturation between 24 and 33^+6^ weeks may be considered depending on the scheduled timing of delivery [51].

In case of successful laser dichorionization and normal ultrasound scans at follow-up, it is reasonable to prolong the pregnancy up to 36–37 weeks of gestation (as for uneventful monochorionic pregnancies) [5].

### 4.8. Atypical TTTS

The clinical presentation of twin-to-twin transfusion syndrome may differ from the typical picture of TTTS, and the progression of the Quintero stages may not be consequential. For example, some cases might present with Doppler abnormalities without having presented with absent bladder of the donor or may have characteristics of disease severity, without the classic diagnostic feature of oligopolyhydramnios [52]. Also, other conditions, such as selective fetal growth restriction (sFGR) or twin anemia polycythemia syndrome (TAPS) may occur with TTTS. These pregnancies may have worse perinatal outcomes compared to the typical form; their management should be individualized, and they should be referred to a tertiary referral centre for maternal–fetal medicine [52].

## 5. Twin Anemia Polycythemia Syndrome (TAPS)

### 5.1. Introduction

This condition was described for the first time by Lopriore et al. in 2007, when they encountered a case of monochorionic twins without volume discordance but with discordance in the hemoglobin levels between the two fetuses [53]. TAPS has an incidence of 1–5% in monochorionic pregnancies, and the optimal surveillance and treatment for this condition are still an object of debate.

In twin anemia polycythemia syndrome, the placental vascular anastomoses have a smaller diameter (<1 mm) than those observed in TTTS that allow for only the slow flow of red blood cells without determining the imbalance of blood flow typical of TTTS. This passage causes hemoglobin discordance, with one “donor” twin who becomes anemic and one “recipient” twin who becomes polycythemic [54].

This complication may occur spontaneously, it can be consequent to laser ablation because of small remaining anastomosis in pregnancies complicated by TTTS, or it may occur with TTTS in the atypical forms of the disease [54].

### 5.2. Diagnosis

The diagnosis of TAPS is often made postnatally (40–60% of cases); an intertwin difference in hemoglobin > 8 g/dL with the anemic neonate also presenting with reticulocytosis (intertwin reticulocyte count ratio > 1.7) [55] or with evidence of small vascular anastomoses in the shared placenta are diagnostic of TAPS. In addition, a discordance in the hematocrit value > 24% might be indicative of TAPS [5,54].

The prenatal diagnosis, according to the current ISUOG guidelines, is based on the findings of middle cerebral artery (MCA) peak systolic velocity (PSV) > 1.5 multiples of median (MoM) for gestational age in the donor and MCA PSV < 1.0 MoM in the recipient, suggesting anemia and polycythaemia, respectively, with high diagnostic accuracy [5,54]. Other additional characteristics may be observed in pregnancies complicated by TAPS, such as different placental echogenicity and thickness, with the donor area appearing thickened and the recipient part appearing thin and echolucent or a “starry sky” appearance of the liver in the recipient due to a diminished echogenicity of liver parenchyma and increased echogenicity of the portal system [5,54].

Nevertheless, recent studies have reported that the intertwin delta MCA PSV > 0.5 multiples of the median (MoM) may be more accurate for diagnosing TAPS compared to the current prenatal diagnostic criteria [56,57,58]. A recent Delphi consensus about the diagnosis and monitoring of TAPS cases stated that the agreement on the diagnostic criteria included the combination of MCA PSV ≥ 1.5 MoM in the donor twin and ≤0.8 MoM in the recipient twin; the alternative diagnostic criteria of MCA PSV discordance ≥ 1 MoM was also proposed [59]. Further studies are needed to validate these diagnostic criteria before being included in the current guidelines.

ISUOG guidelines recommend performing ultrasound screening for TAPS every two weeks, with MCA PSV Doppler measurements starting from 20 weeks of gestation [5]. In cases of diagnosed TAPS, the follow-up schedule proposed in the Delphi consensus included weekly ultrasounds [59].

The prenatal classification system of the disease is based on five stages of severity is shown below (Table 3).

### 5.3. Outcomes and Management

The outcomes of TAPS may vary from intrauterine fetal demise to the delivery of two healthy babies with discordant hemoglobin levels. Severe brain damage may occur in cases of severe anemia.

In a large cohort of cases of spontaneous TAPS, the authors reported a 5% rate of fetal demise and a 4% rate of neonatal mortality, with the donor having a four times higher risk of neurodevelopmental delay compared to the recipient [60]. A recent meta-analysis about the outcomes of spontaneous TAPS and TAPS occurring after laser-treated TTTS showed that intrauterine fetal demise occurred in 5.2% of cases with spontaneous TAPS and in 10.2% of cases in post-laser TAPS, and neonatal death occurred in 4% and 9.2% of cases, respectively [61].

The optimal treatment of this condition remains controversial; the current guidelines suggest individualizing the management of single cases considering the stage of the disease, the gestational age at diagnosis, comorbidities, parental choice, and the feasibility of intrauterine treatment [5].

The treatment options include laser ablation, selective termination of one twin, intrauterine transfusion of the anemic twin and partial exchange transfusion of the recipient twin, and conservative management [5].

If conservative management is adopted, experts suggest a follow-up with ultrasound assessment once a week, which must be intensified if additional anomalies are encountered. This approach may be considered in the first and second stage of the disease [62].

Intrauterine transfusion with the partial exchange transfusion of the recipient aims to increase levels of hemoglobin in the donor and reduce hyperviscosity due to polycythemia in the recipient. It is performed with a 21G needle inserted in the umbilical cord of both twins, where 3–5 mL of blood is taken from the recipient and replaced with saline solution; in the donor, the amount of blood that is transfused is calculated considering the hematocrit. Intraperitoneal transfusion may also be considered [62].

Fetoscopic laser ablation and selective fetal termination may be performed in twin-to-twin transfusion syndrome. The very small anastomosis may cause failure of the laser ablation; therefore, the Solomon technique should be performed [62].

The comparison of different treatment strategies for TAPS showed no significant difference in terms of morbidity and mortality [61]. In particular, intrauterine demise occurred in 9.8% of cases of expectant management, 13.1% in cases treated with laser surgery, 12.1% with intrauterine transfusion, and 7.6% with selective termination [61]. Also, the rate of severe neonatal morbidity was similar between the treatment groups [61].

In the case of early presentation of the disease, fetal surgery should be considered since it is the only potential definitive treatment, whereas when TAPS occurs in late pregnancy, intrauterine transfusion should be considered as a therapy of support until delivery [62].

Neurodevelopmental delay may occur in pregnancies complicated by TAPS in up to 20% of cases. ISUOG guidelines recommend performing an accurate study of the brain with neurosonography and MRI in the third trimester and in the first years of infancy [5].

## 6. Twin Reversed Arterial Perfusion Sequence (TRAP)

TRAP complicates 2.6% of all monochorionic or diamniotic twin pregnancies with an incidence of 1 in 9500–11,000 pregnancies [63]. It is characterized by the presence of an acardiac twin perfused by a structurally normal twin (“pump twin”); this perfusion occurs through a reversed arterio-arterial anastomosis within the placenta, which delivers deoxygenated blood via a retrograde flow [63]. The acardiac twin returns deoxygenated blood to the pump twin through a veno-venous anastomosis. This condition may cause heart failure of the pump twin because of the high-output circulation, including issues such as cardiomegaly, tricuspid regurgitation, altered umbilical artery and ductus venosus Dopplers, polyhydramnios, and hydrops [63]. The mortality of the pump twin reaches 85% if untreated [63]. The diagnosis is usually made with ultrasound in the case of different biometrical measurements of the twins, with one twin presenting with absence of a cardiac structure or with a rudimental cardiac structure, usually associated with other malformations in the head (acranial), upper and lower extremities, and subcutaneous edema [64]. The colour Doppler demonstrates a reversed blood flow in the umbilical artery of the acardiac twin [64].

The management options of TRAP include expectant management or fetal intervention with cord occlusion of the acardiac twin [5,65]. Some studies have investigated the role of possible predictors of adverse outcomes in TRAP. Wong et al. proposed the abdominal circumference ratio between the acardiac and the pump twin combined with signs of cardiac compromise in the pump twin as possible indicators for guiding management (expectant management or prompt intervention) [64]. Similarly, other authors investigated the role of the pump/acardiac twin umbilical venous diameter (UVD) ratios as a possible indicator for adverse outcomes; it was shown that complicated cases had decreased UVD ratios compared to uncomplicated cases [66].

Recent evidence supports fetal intervention rather that expectant management, especially in cases with signs of cardiac compromise of the pump twin or other poor prognostic indicators, such as polyhydramnios, a large size of acardiac mass, and monoamniotic twin pregnancy [67].

The goal of intrauterine treatment is to interrupt the blood flow from the pump twin toward the acardiac mass. Treatment options include bipolar cord coagulation guided by ultrasound or fetoscopy, radiofrequency ablation, microwave ablation, laser ablation of the umbilical cord, or interstitial laser ablation [67].

In utero treatment is associated with a significant improvement of the perinatal survival of the pump twin (80–92%) [68], but the timing of the intervention is still an object of study. A review about selective termination of pregnancy in MC twins included cases of TRAP and reported better outcomes with the procedure performed after 18 weeks of pregnancy; however, intrafetal laser treatment was not included in the analysis [24]. On the other hand, another meta-analysis about the intrafetal laser treatment in TRAP cases reported better outcomes when the procedure was performed before 16 weeks of gestation [68]. A multicenter randomized controlled study about the optimal timing for intervention in TRAP is now ongoing, comparing 13–15 weeks versus after 16 weeks of pregnancy (“TRAPIST Trial”; NCT02621645).

## 7. Selective Fetal Growth Restriction (sFGR)

This condition affects 10–15% of monochorionic twin pregnancies, and it is associated with increased perinatal morbidity and mortality. It is characterized by the presence of one twin significantly smaller for their gestational age and compared to the other twin, who is adequate for their gestational age. The most accredited pathogenetic theory of sFGR is the unequal sharing of the unique placenta between the two twins; it is not infrequent to observe a velamentous insertion of the cord blood in the small twin [69,70].

The diagnostic criteria reported on the ISUOG guidelines for sFGR in monochorionic pregnancies include the presence of the estimated fetal weight (EFW) of one twin < 10th percentile plus the intertwin EFW discordance >25% [5]. However, this topic is an object of discussion in the current literature. The Delphi consensus regarding the diagnosis of sFGR resulted in an agreement on new diagnostic criteria, shared by a panel of experts, with the EFW < 3rd percentile of one twin or two criteria out of four of the following: EFW of one twin < 10th percentile; abdomen circumference (AC) of one twin < 10th percentile; intertwin EFW discordance ≥ 25%; and abnormal umbilical artery Doppler of the small twin (pulsatility index PI > 95th percentile) [71]. Discordance in the amount of amniotic fluid is not typical of sFGR.

The standard classification of severity for sFGR in monochorionic twins is the Gratacos classification, which relies on the pattern of the umbilical artery Doppler in the smaller twin, with three types characterized by different prognosis [72] (Table 4). Type I sFGR is associated with the best prognosis, with a low risk of progression (16%) or fetal demise of the small twin [73]. Type II sFGR has the worst prognosis, with a high risk of progression (59%) and fetal demise with possible consequences regarding the co-twin, such as brain damage or fetal demise [73]. Type III sFGR is characterized by an intermittent pattern, with a 10% risk of progression and unpredictable outcomes [73].

Selective fetal growth restriction in MC twin pregnancies can be classified as early or late when it occurs before or after 24 weeks of gestation, as the cut-off of 32 weeks used in singletons would be nearly the time of delivery in these pregnancies [5]. A recent cohort study about monochorionic diamniotic pregnancies complicated by sFGR reported that early sFGR presented more frequently with Type II sFGR (15.4%), which has the worst prognosis, whereas late sFGR presented with fewer Type II cases (5.6%) [74]. Moreover, the risk of superimposed TTTS was higher in the early sFGR group compared to the late group (26.9% vs. 5.6%) [74].

The main predictors of adverse perinatal outcomes, particularly fetal demise, were shown to be the gestational age at presentation (early sFGR), ductus venosus, and umbilical artery Dopplers [75]. The Gratacos classification, however, does not take into account all these predictors; a new classification system for sFGR in monochorionic pregnancies has been recently proposed to include all possible predictors of adverse perinatal outcomes (Table 5) [76].

The ultrasound surveillance of sFGR in monochorionic pregnancies requires weekly ultrasound scans for Doppler assessment [5].

The management options in sFGR include expectant management, the fetoscopic laser ablation of placental anastomosis, or selective termination of the restricted fetus by cord occlusion [5]. The quality of evidence about this topic is low and no randomized controlled trials have been conducted [77,78,79]; therefore, there are no recommendations regarding the most appropriate management in these cases and the choice should be individualized per single case, taking into account the characteristics of the disease and the parent’s wishes. A recent meta-analysis investigated the outcomes of pregnancies complicated by sFGR in different stages according to the management; intrauterine demise occurred more frequently in the laser group in all stages of the disease (Gratacos Type I, II, and III); the cord occlusion group was associated with lower rates of demise, but the technique itself included a selective termination [80]. In Type I, the expectant management was associated with a very low risk of fetal demise (3.1%, with 97.9% of intact survival), so it should be considered in the decision-making at this stage [80]. The overall morbidity, on the other hand, was lower in the laser group compared to the selective termination and the expectant management groups [77,78,79,80].

In conclusion, since there is not strong evidence on the optimal treatment for these particular cases, management should be discussed with the parents and individualized based on the staging of the disease and the gestational age at diagnosis.

The timing of delivery in cases of sFGR in monochorionic pregnancy should be individualized for each case, considering the fetal well-being, fetal growth, Dopplers, and computerized CTG assessments [5]. Because of the lack of specific guidelines regarding the management of twin pregnancies according to CTG assessments, clinicians interpret it according to standards defined for singletons.

## 8. Single Fetal Demise

In the case of single fetal demise after the first trimester of pregnancy, it has been widely reported in the current literature that the surviving co-twin may develop brain damage (up to 26% of cases) [81,82]. The underlying mechanisms of the brain damage in the surviving twin are still debated; some authors stated that coagulopathy and infarctions in the cerebral tissue may be the cause of the damage [83]; others reported that the acute hemodynamic imbalance caused by the sudden loss of cardiac activity in the demised fetus may affect the survivor twin because of the placental vascular anastomosis, causing a transient cerebral reduced perfusion with possible ischemic brain injury [84]. The most frequent diagnosis of brain damage in these cases are hemorrhagic or ischemic focal lesions regarding the basal ganglia, thalamus, cortex, multicystic encephalomalacia, microcephaly, porencephaly, and hydrocephaly. The risk of brain damage is higher in the case of demise at late gestational ages [84,85]. In the case of pregnancies complicated by TTTS, single fetal demise is associated with a higher risk of developing brain injuries [86].

Long-term neurodevelopmental impairment is not always consequent to brain damage; a meta-analysis on this topic reported a prevalence of neurodevelopmental delay of 26%, with a prevalence of brain damage of 34% [87].

It is mandatory to offer proper counselling to the parents about the risks of developing neurodevelopmental sequelae in these cases. Neuroimaging achieved with targeted neurosonography and magnetic resonance imaging plays an essential role in the detection of brain involvement in twin pregnancies complicated by single demise [83,87]. However, even in case of normal imaging, the risk of neurodevelopmental impairment may not be ruled out. Further studies are needed regarding the residual risk of long-term impairment in the case of normal findings at prenatal imaging.

## 9. Conclusions

In conclusion, monochorionic pregnancies are considered high-risk pregnancies because of the higher morbidity and mortality compared to singleton or dichorionic twin pregnancies. The management of these pregnancies require high levels of expertise in the field of fetal medicine, and counselling the couples with monochorionic pregnancies about possible complications and management is of great importance in routine clinical practice.

## Figures and Tables

**Table 1:** MCDA complications and their diagnostic criteria.

Ultrasound Parameters	Diagnostic Criteria	Complications
Amniotic fluid discordance	DVP < 2 cm and >8 (<20 weeks) or >10 (>20 weeks) cm	Twin-to-twin transfusion syndrome (TTTS)
Fetal biometry and Doppler	-EFW < 3rd centile in one twin OR >2 criteria among -EFW and/or AC < 10th percentile -UA PI > 95th percentile -EFW discordance > 25%	Selective fetal growth restriction (sFGR)
MCA-PSV	MCA-PSV > 1.5 MoM in one twin and <1 MoM in the other	Twin anemia polycythemia syndrome (TAPS)

DVP = deepest vertical pocket; EFW = estimated fetal weight; AC = abdominal circumference; UA = umbilical artery; PI = pulsatility index; MCA = middle cerebral artery; PSV = peak systolic velocity; MoM = multiple of medians.

**Table 2:** Quintero staging system.

Stage	
Stage I	Polyhydramnios–oligohydramnios
Stage II	No visualization of the fetal bladder in the donor twin
Stage III	Doppler abnormalities Absent/reversed umbilical artery end-diastolic flow Reversed a wave flow in the ductus venosus Pulsatile umbilical vein flow
Stage IV	Fetal hydrops in one or both twins
Stage V	Fetal demise of one or both twins

**Table 3:** Classification system for TAPS.

Stage of the Disease	Antenatal Staging: Ultrasound Criteria	Postnatal Staging: Intertwin Hb Difference
Stage I	Donor MCA-PSV > 1.5 MoM and recipient MCA-PSV < 1.0 MoM, without other signs of fetal compromise	>8 g/dL
Stage II	Donor MCA-PSV > 1.7 MoM and recipient MCA-PSV < 0.8 MoM, without other signs of fetal compromise	>11 g/dL
Stage III	Stage 1 or 2 and cardiac compromise in the donor or in the recipient (UA-AREDF, UV pulsatile flow, or DV increased or reversed flow)	>14 g/dL
Stage IV	Hydrops of the donor	>17 g/dL
Stage V	Death of one or both fetuses	>20 g/dL

AREDF, absent or reversed end-diastolic flow; DV, ductus venosus; Hb, hemoglobin; MCA, middle cerebral artery; MoM, multiples of median; PSV, peak systolic velocity; UA, umbilical artery; UV, umbilical vein.

**Table 4:** Gratacos classification for sFGR in monochorionic twins.

Type	Doppler Pattern
Type I	Normal umbilical artery Doppler in the small twin (positive end-diastolic flow)
Type II	Persistent absent or reversed end-diastolic flow in umbilical artery Doppler in the small twin
Type III	Intermittent absent or reversed end-diastolic flow in umbilical artery Doppler in the small twin

**Table 5:** New proposed classification for sFGR in monochorionic pregnancies.

Stage	Ultrasound Findings
Stage I	Normal umbilical artery Doppler in the small twin (positive end-diastolic flow)
Stage IIa	Persistent absent or reversed end-diastolic flow in umbilical artery Doppler in the small twin
Stage IIb	Intermittent absent or reversed end-diastolic flow in umbilical artery Doppler in the small twin
Stage III	Abnormal ductus venosus in the small twin
Stage IV	Superimposed TTTS
Stage V	Intrauterine demise of the small twin
